# Identification of the Ferroptosis-Related Long Non-Coding RNAs Signature to Improve the Prognosis Prediction in Papillary Renal Cell Carcinoma

**DOI:** 10.3389/fsurg.2022.741726

**Published:** 2022-03-04

**Authors:** Xinfang Tang, Feng Jiang, Xiaoyu Wang, Ying Xia, Yan Mao, Yan Chen

**Affiliations:** ^1^Department of Nephrology, The Affiliated Lianyungang Oriental Hospital of Xuzhou Medical University, The Affiliated Lianyungang Oriental Hospital of Kangda College of Nanjing Medical University, The Affiliated Lianyungang Oriental Hospital of Bengbu Medical College, Lianyungang, China; ^2^Department of Neonatology, Obstetrics and Gynecology Hospital of Fudan University, Shanghai, China; ^3^Department of Pediatrics, The Second Affiliated Hospital of Nantong University, Nantong, China; ^4^Department of Pediatrics, The First Affiliated Hospital of Nanjing Medical University, Nanjing, China; ^5^Department of Nephrology, Jiangsu Province Geriatric Hospital, Jiangsu Province Official Hospital, Nanjing, China

**Keywords:** ferroptosis, lncRNA, immune environment, prognosis, papillary renal cell carcinoma

## Abstract

Papillary renal cell carcinoma (pRCC) is one of the epithelial renal cell carcinoma (RCC) histological subtypes. Ferroptosis is a new iron-dependent form of cell death that has been seen in a variety of clinical situations. Using differentially expressed ferroptosis-related long non-coding RNAs (lncRNAs) from patients with pRCC in The Cancer Genome Atlas; we built a prognostic lncRNA-based signature. We discovered seven different lncRNAs that were strongly linked to the prognosis of patients with pRCC. High-risk scores were linked to a poor prognosis for pRCC, which was confirmed by the findings of Kaplan–Meier studies. In addition, the constructed lncRNA signature has a 1-year area under the curve (AUC) of 0.908, suggesting that it has a high predictive value in pRCC. In the high-risk group, Gene set enrichment analyses (GSEA) analysis identified immunological and tumor-related pathways. Furthermore, single-sample GSEA (ssGSEA) revealed significant differences in T cell functions checkpoint, antigen presenting cell (APC) co-stimulation, inflammation promoting, and para inflammation between the two groups with different risk scores. In addition, immune checkpoints like PDCD1LG2 (PD-L2), LAG3, and IDO1 were expressed differently in the two risk groups. In summary, a novel signature based on ferroptosis-related lncRNAs could be applied in predicting the prognosis of patients with pRCC.

## Introduction

Renal cell carcinoma (RCC) is classified into three types according to the clinically pathological type: chromophobe RCC (chRCC), papillary RCC (pRCC), and clear cell RCC (ccRCC) (1). pRCC is a malignant tumor of the renal parenchyma (2), and it is the second most common RCC subtype, for it accounts for 15–20% of all kidney tumors (3, 4). The 5-year overall survival (OS) rate of localized pRCC has been reported as 78–79% (5). Despite the fact that many individuals have quite varied treatment outcomes and prognoses, their tumor types are all the same histologically (6). While individuals with advanced pRCC unfortunately have no viable therapy options nowadays. A lack of reliable biomarkers for early cancer detection or suboptimal preclinical models has hampered effective clinical treatment of pRCC. Currently, the majority of anti-tumor medicines used in clinic work by triggering the death of cancer cells. Hence, it is important to look into alternative types of cell death to solve the resistance of tumor cells while also finding novel and effective prognostic biomarkers for pRCC.

In the last several decades, there have been more and more researches exploring ferroptosis in tumors. Unlike apoptosis and autophagy, ferroptosis is actually a type of cell death dependent on iron and depends on intracellular buildup of reactive oxygen species (ROS) (7). Iron metabolism dysregulation has been reported to be a risk factor for many neoplasms, and plays a role in the development of tumors. Meanwhile, treatment with iron chelators could reverse the death phenotype of cancer cells, suggesting that cell death is iron-dependent (8). Cancer cells, in comparison to normal cells, have iron addiction, or an excessive reliance on iron for proliferation (9). Resistance to chemotherapeutic treatments is widely recognized to be a significant cause of mortality in cancer patients (10). Indeed, stimulation of ferroptosis pathways might be able to overcome existing chemotherapeutic drug resistance (11), bringing up a new therapeutic area for cancer therapy. Long non-coding RNAs (lncRNAs) actually belong to a subgroup of RNA molecules, which characterizes with a length of 200 nucleotides, and could regulate gene expression levels (12). In addition to gene regulation, lncRNA is also involved in a variety of biological regulatory mechanisms, which play important roles in tumor incidence, development, and metastasis (13). Emerging studies pointed out lncRNA is important for the regulation of ferroptosis (14). While only few researches have been conducted on ferroptosis-related lncRNAs. In lung cancer, the lncRNA P53RRA has been shown that could promote ferroptosis (15). The silence of lncRNA ZFAS1 was found to suppress ferroptosis, along with the reduction of inflammation and lipid peroxidation (16).

However, there have still been few sequence-based studies systematically evaluating the characteristics of lncRNAs related to ferroptosis and their relationships with the clinical OS of patients with PpRCC. Here, using the pRCC information extracted from The Cancer Genome Atlas (TCGA) database, we built a risk model composed of several ferroptosis-related lncRNAs, which were found closely related with the prognosis of patients with pRCC. The predictive significance of the constructed signature was further investigated by ferroptosis-related mRNA roles, and the immunological response in patients with pRCC. This ferroptosis risk model may provide more intuitive and rational information for physicians to treat patients with pRCC in the future.

## Methods

### Collection of Data

We downloaded the data of RNA-sequence of 321 samples (such as 32 normal and 289 tumors) from the TCGA-pRCC database. The ferroptosis-related genes were extracted from FerrDb (17) (http://www.zhounan.org/ferrdb/), which is actually a manually curated, comprehensive, and up-to-date web-based database that provided information of ferroptosis markers, ferroptosis regulators, and associated diseases. We extracted a total of 382 ferroptosis-related genes from FerrDb, such as 150 drivers, 123 markers, and 109 suppressors (Supplementary Table 1). Pearson's correlation analysis was used to evaluate the association between the identified ferroptosis-related lncRNAs and pRCC. If the correlation coefficient |R| was <0.4 at *p* < 0.001, the relationship was deemed significant. The differentially expressed gene (DEG) analysis was applied with the “Limma” package on TCGA-pRCC transcriptome file, which finally identified 56 DEGs. An empirical Bayesian method was applied to estimate the fold change between tumor samples and control samples using moderated *t*-tests. The adjusted *p*-value for multiple testing was calculated using the Benjamini–Hochberg correction. The genes with an absolute log2 fold change >1 and adjusted *p* < 0.05 were identified as DEGs. Clinical data such as gender, age, stage, TMN, and treatment were retrieved from TCGA-pRCC, as well as the information of samples' survival status and survival time, which were shown in Supplementary Table 2. False discovery rate (FDR) <0.05 and |log_2_FC| ≥1 was used to identify the significantly differentially expressed ferroptosis-related lncRNAs.

### Gene Ontology and Kyoto Encyclopedia of Genes and Genomes Enrichment Analyses

We explored the function of DEG related to ferroptosis *via* Gene Ontology (18) (GO, http://www.geneontology.org/) and Kyoto Encyclopedia of Genes and Genomes (19) (KEGG, http://www.kegg.jp/) analyses using R software (version 4.0.5). A significance level of *p* < 0.05 was deemed significant.

### Construction of the Prognostic Risk Model According to the Identified Ferroptosis-Related lncRNAs

The ferroptosis-related lncRNAs selected by LASSO-penalized Cox regression and univariate Cox regression analyses were subsequently entered into the multivariate Cox regression analysis. We then constructed a risk model based on the identified ferroptosis-related lncRNAs as follows:


$$\begin{array}{l} Riskscore=\overset{n}{\underset{i=1}{\sum }}Exp{\left(i\right)}^{*}Coe\left(i\right) \end{array}$$

The Exp(i) represented the expression levels of the ferroptosis-related lncRNAs while the Coe(i) represented the corresponding coefficient. The risk score for each patient with pRCC was evaluated, the median one of which was identified as the cutoff to divide the patients into a low-risk group (with risk score < median score) or high-risk group (with risk score ≥ median score).

### Gene Set Enrichment Analyses and the Predictive Nomogram

The KEGG enrichment pathways were explored by the gene set enrichment analyses (GSEA) software (20) (http://www.broadinstitute.org/gsea/) to define signatures of the lncRNAs, which were subsequently searched in the pRCC data from TCGA. A nomogram was created by combining the prognostic signals to predict the 1-, 3-, and 5-year clinical OS of patients with pRCC.

### Immunity Analysis

We used the Tumor Immune Estimation Resource (TIMER) (21), CIBERSORT (22), QUANTISEQ (23), XCELL (24), MCP counter (25), and Estimating the Proportion of Immune and Cancer cells (EPIC) (26) to comparatively assess cell immune responses or cellular components between two different risk groups. Moreover, single-sample GSEA (ssGSEA) was utilized to compare and quantify the subgroups of the tumor-infiltrating immune cells, as well as their immunological function, in two groups with different risks. Previous research yielded a list of possible immunological checkpoints.

### Statistical Analysis

Bioconductor packages were used to analyze the data in R software (version 4.0.5). Normally distributed data were analyzed by using the unpaired student's *t*-test, while non-normally distributed data were analyzed by using the Wilcoxon test. To find the differentially expressed lncRNAs, we used Benjamini–Hochberg method based on the FDR. Using “GSVA” (R-package) (27), we compared the ssGSEA-normalized pRCC DEG with a genome. To further compare the sensitivity and specificity of the generated prognostic signature with those of other clinicopathological characteristics, we performed the receiver operating characteristic (ROC) and decision curve analysis (DCA) (28). A visual co-expression network of mRNA-lncRNAs was constructed using the ggalluvial R software package. Using the logistic regression analysis, the connection between the identified ferroptosis-related lncRNAs and the chosen clinicopathological symptoms was investigated and shown in a heat map graph. Based on the ferroptosis-related lncRNAs risk model, the Kaplan–Meier survival analysis was carried out to evaluate the prognosis of patients with pRCC. Statistical significance was established at *p* < 0.05 for each analysis.

## Results

### Enrichment Analyses for the Identified Ferroptosis-Related Genes

**Figure 1:**
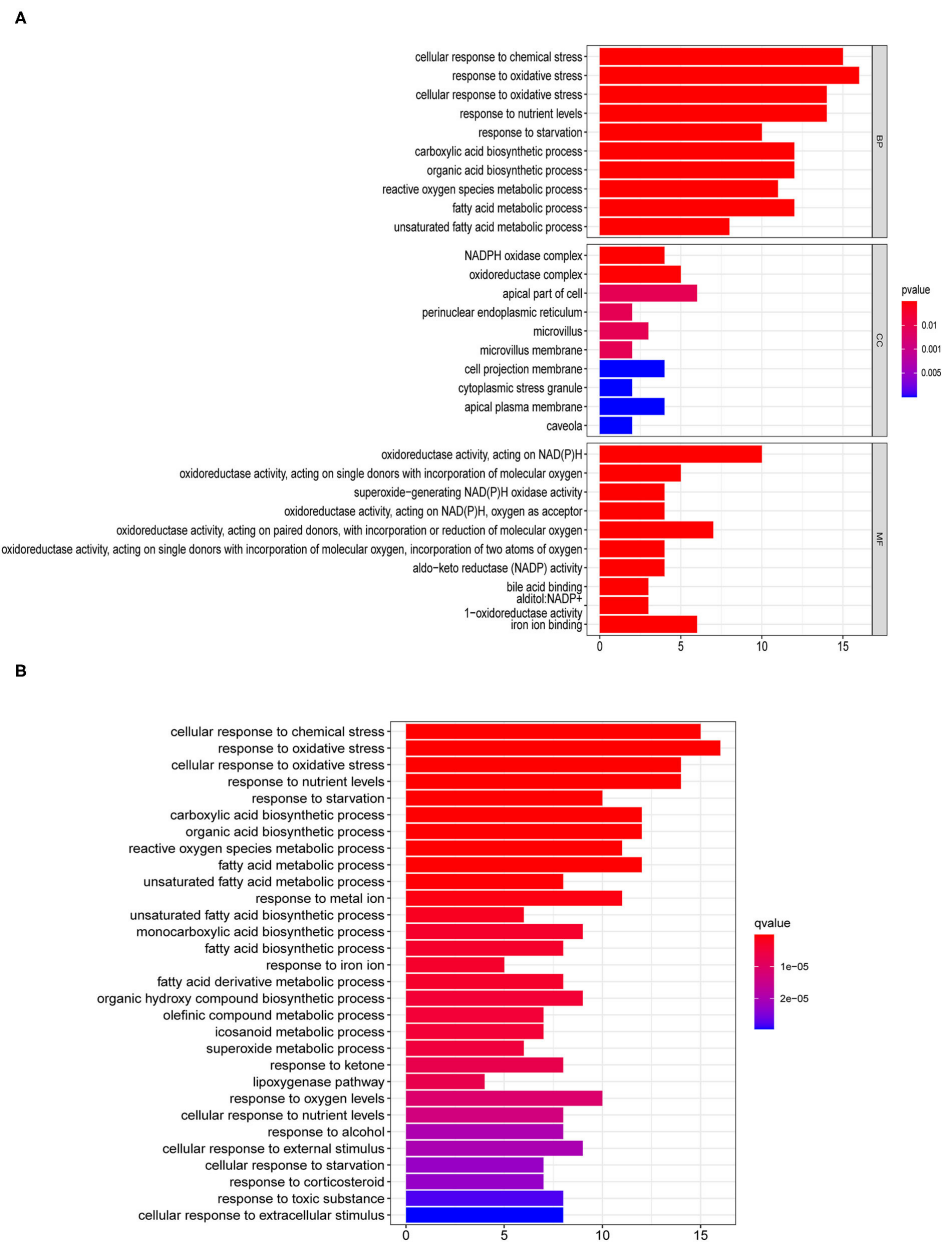
Enrichment pathway analyses for differentially expressed genes (DEG) related to ferroptosis. **(A)** Gene ontology (GO) and **(B)** Kyoto Encyclopedia of Genes and Genomes (KEGG) analyses of differentially expressed ferroptosis-related genes.

We identified a total of 56 DEGs related to ferroptosis (Supplementary Table 3). The GO enrichment pathways mainly included a response to oxidative stress, NADPH oxidase complex, fatty acid metabolic process, iron ion binding, and so on (Figure 1A). KEGG enrichment analysis showed that the over-expressed genes mainly participated in cellular responses to chemical stress, response to oxidative stress, response to iron ion, response to oxygen levels, and so on (Figure 1B).

### The Construction of the Ferroptosis-Based lncRNAs Prognostic Risk Model

**Figure 2:**
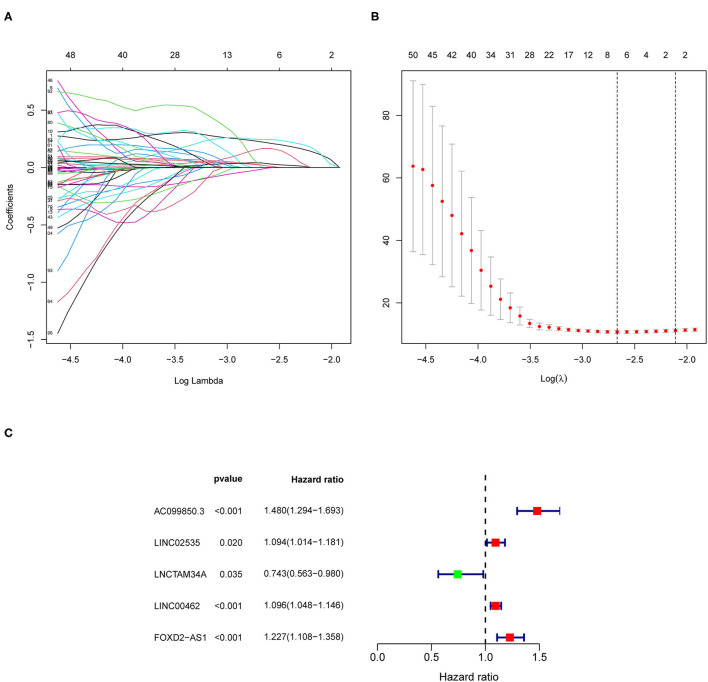
The construction of the ferroptosis-related long non-coding RNAs (lncRNAs) prognostic risk model. **(A,B)** Lasso Cox regression analyses for the ferroptosis-based lncRNAs according to the results of univariate Cox regression analysis. **(C)** Multivariate Cox regression analysis for the ferroptosis-associated lncRNAs.

Based on the information of patients with pRCC from TCGA, we uncovered 945 ferroptosis-related lncRNAs which were differentially expressed between patients and control ones (Supplementary Table 4). Univariate Cox analysis based on the expression level of lncRNAs and the OS of patients with pRCC was carried out to explore the ferroptosis-related lncRNAs (Supplementary Table 5), which were further analyzed by lasso cox regression analysis. Then, 7 lncRNAs associated with ferroptosis were found closely relative to the outcome of patients with pRCC (Figures 2A,B). By multivariate COX analysis, 5 differently expressed lncRNAs (AC099850.3, LINC02535, LNCTAM34A, LINC00462, and FOXD2-AS1) were found as independent prognostic factors of patients with pRCC (Figure 2C and Table 1). We constructed a prognostic signature according to the five differently expressed lncRNAs. The risk score developed was as followed: risk score = 0.39 × AC099850.3 + 0.09 × LINC02535 + 0.09 × LINC00462 + 0.20 × FOXD2-AS1 – 0.30 × LNCTAM34A. We calculated risk scores for samples of pRCC from TCGA, and the median score was chosen as the cutoff. The patients with pRCC were then separated into high-risk group or low-risk group according to their risk scores.

**Table 1:** The 5 differently expressed long non-coding RNAs (lncRNAs) closely related with the overall survival (OS) of papillary renal cell carcinoma (pRCC) patients based on the multivariate Cox regression analysis.

**Gene Id**	**Coef**	**HR**		***P***
AC099850.3	0.391978416	1.479905769		<0.001
LINC02535	0.090129628	1.094316129		0.02
LNCTAM34A	−0.297485468	0.742683376		0.035
LINC00462	0.091654887	1.095986518		<0.001
FOXD2-AS1	0.204445064	1.226844056		<0.001

### Prognostic Value of the Identified Ferroptosis-Related lncRNAs Signature in PRCC

**Figure 3:**
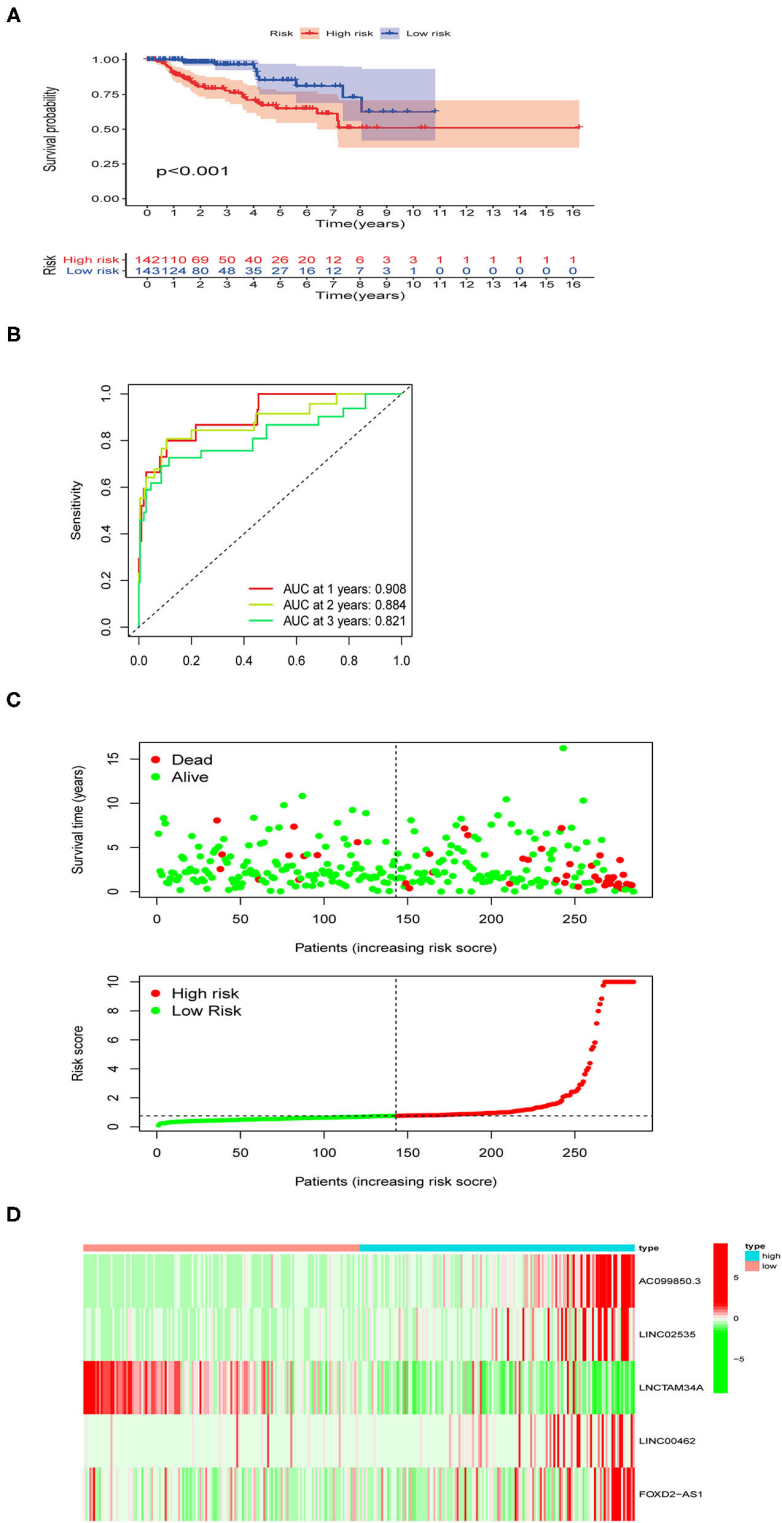
Performance evaluation of the identified risk signature in papillary renal cell carcinoma (pRCC). **(A)** Kaplan–Meier overall survival (OS) curve for patients with pRCC divided into the high- and low-risk groups. **(B)** Receiver operating characteristic (ROC) curves exhibiting the predictive efficiency of the ferroptosis-related lncRNAs-based risk model. **(C)** Risk survival status plot in the high- and low-risk groups. **(D)** A heatmap showing the five identified ferroptosis-based lncRNAs expression profiles in two risk pRCC groups from The Cancer Genome Atlas (TCGA) database.

As shown in Figure 3A, the survival time of patients was shorter in the high-risk pRCC group than in the low-risk pRCC group (*p* < 0.001). Also, the AUC value of 1-, 3-, 5-year survival rate for the constructed signature based on the ferroptosis-related lncRNAs was 0.908, 0.884, and 0.821, respectively (Figure 3B). According to the results of the risk survival status plot for patients with pRCC, it could be easily found that the risk score was inversely related to the survival of patients with pRCC (Figure 3C). Moreover, the heatmap in Figure 3D showed that most of the identified ferroptosis-related lncRNAs were positively correlated with our risk model.

**Figure 4:**
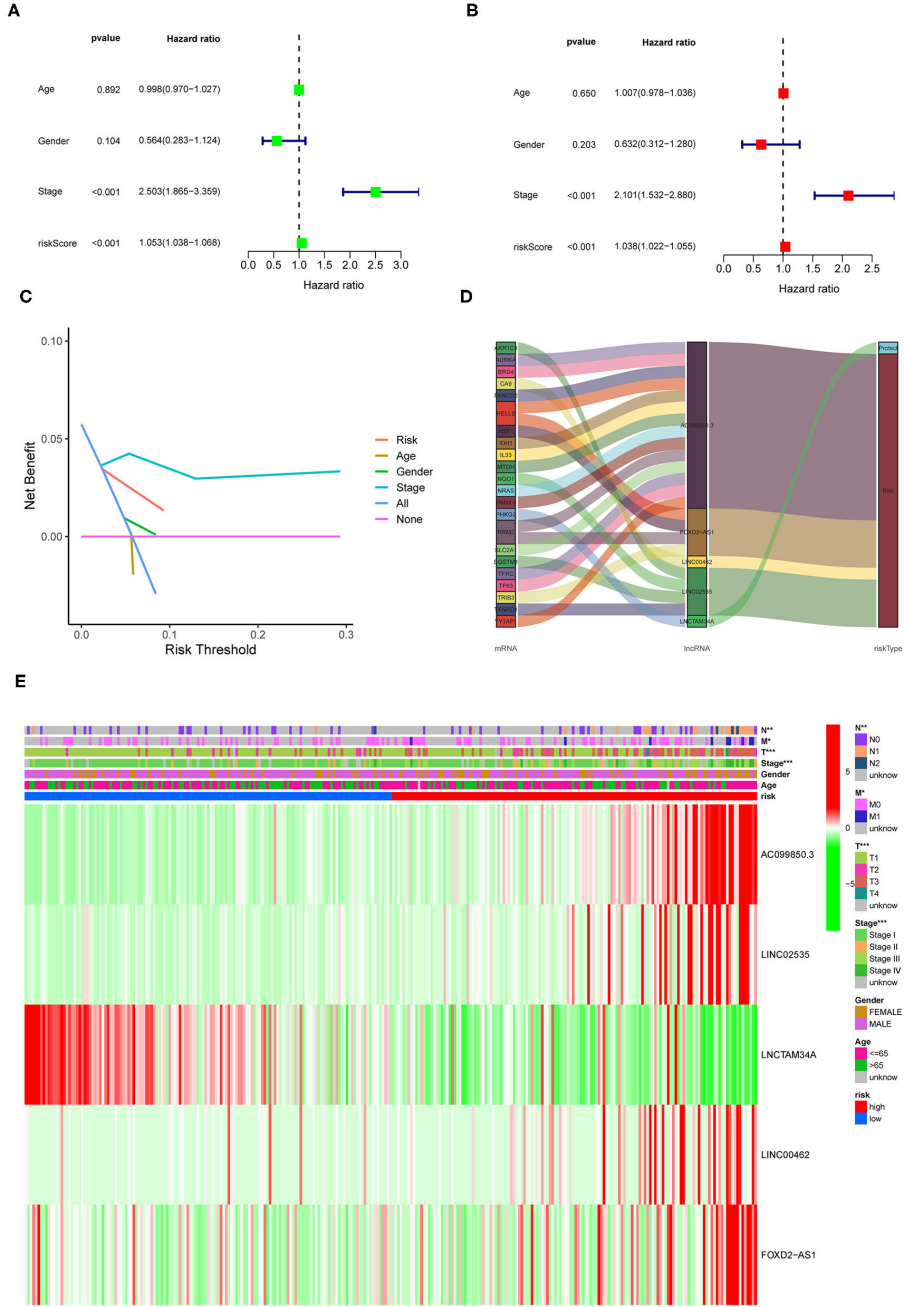
Prognostic value of the identified signature in pRCC. **(A,B)** Univariate and multivariate COX regression analyses for the expression levels of ferroptosis-related lncRNAs. **(C)** The decision curve analysis (DCA) results for the risk factors. **(D)** Sankey diagram showing the relationship between the identified lncRNAs and mRNAs. **(E)** A heatmap for the identified ferroptosis-related lncRNAs in the prognostic risk model, along with the clinicopathological features.

**Figure 5:**
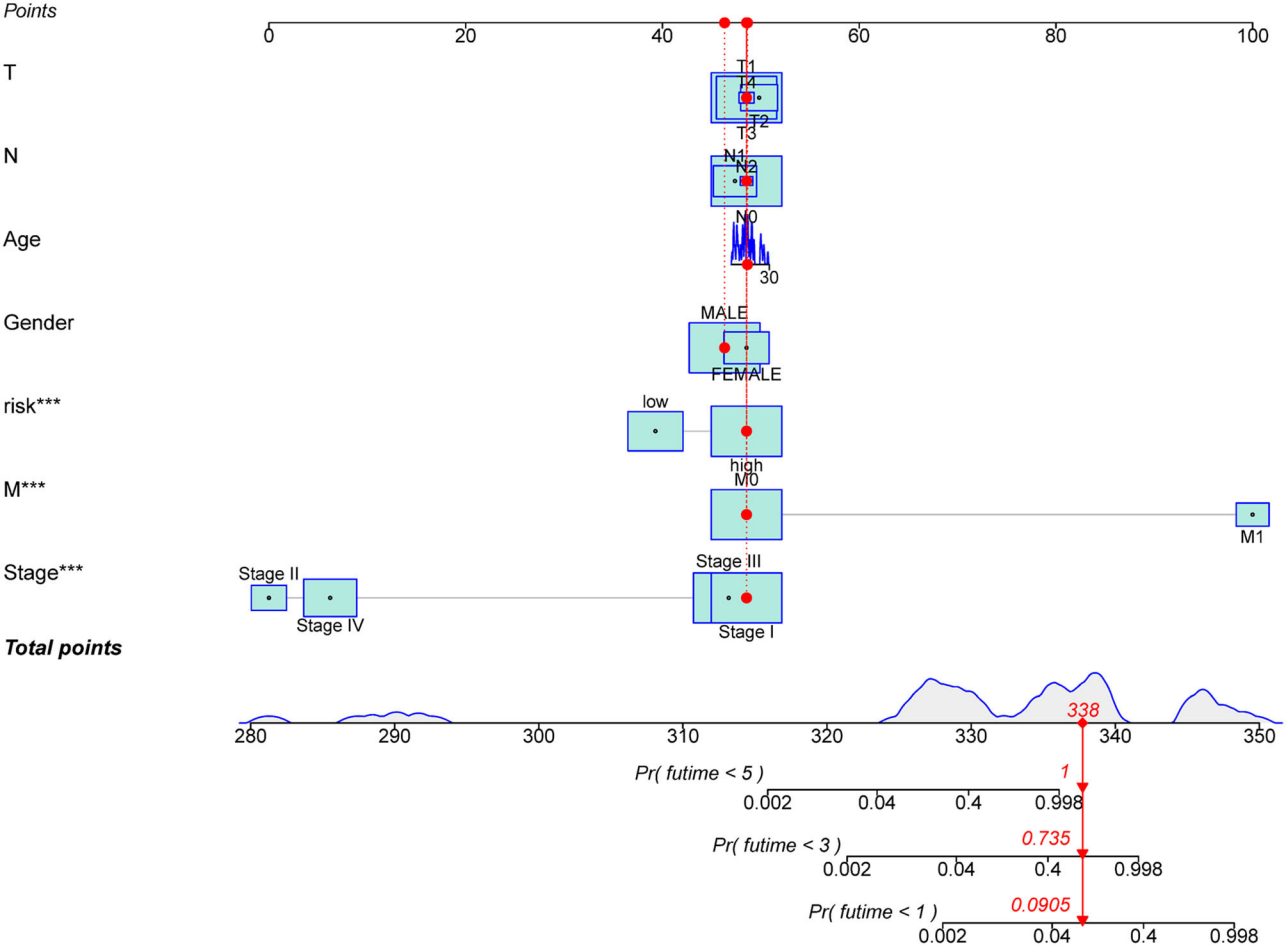
A nomogram for the ferroptosis-related lncRNAs with prognostic value and the clinicopathological factors of patients with pRCC. ***p < 0.001.

To investigate the prognostic value of the newly constructed signature in predicting the OS of patients with pRCC, we further conducted univariate and multivariate Cox regression analyses, which revealed that the risk model was an independent factor with prognostic value for predicting the OS of patients with pRCC (Figures 4A,B). The DCA result also confirmed that the signature exhibited a nice performance in predicting the prognosis of pRCC (Figure 4C). The relationship between mRNA and these 5 identified ferroptosis-related lncRNAs was shown in Figure 4D. In addition, we analyzed the relationship between the clinicopathological manifestations of patients with pRCC and the prognostic risk model (Figure 4E). According to the outcome of clinical management, we divided the patients into a remission group (such as 126 samples) and a non-remission group (such as 16 samples). As shown in Supplementary Figure 1, patients in the non-remission group had significantly higher risk scores than those in the remission group. Supplementary Figure 2 further showed that AC099850.3, LINC02535, and FOXD2-AS1 were significantly higher in the non-remission group than in the remission group, while LNCTAM34A was highly expressed in the remission group. LINC00462 was not significantly different between the two groups, but this may be due to the small sample size. The hybrid nomogram (Figure 5), which combined clinicopathological features and the newly constructed ferroptosis-related lncRNAs-based risk model, was stable and accurate. Hence, it might be used in clinic to promote the management of patients with pRCC.

Cancer-specific survival is also known as disease-specific survival (DSS) (29). To further explore the prognostic value of the constructed risk model, we carried out survival analyses separately based on the OS and the DSS of patients with pRCC. According to the expression levels of each of the identified five lncRNAs, 289 samples were divided into two groups. Then we explored the difference in survival time between groups. As shown in Supplementary Figure 3, patients in the AC099850.3, LINC02535, LINC00462, or FOXD2-AS1 highly expressed groups had significantly lower OS and DSS than patients in the low expression group, while the opposite was the case for LNCTAM34A. The trends of these results were consistent with the coefficients of the five lncRNAs in the prognostic risk model, indicating that our risk models perform better in predicting both OS and DSS in patients with pRCC.

### GSEA Analyses

**Figure 6:**
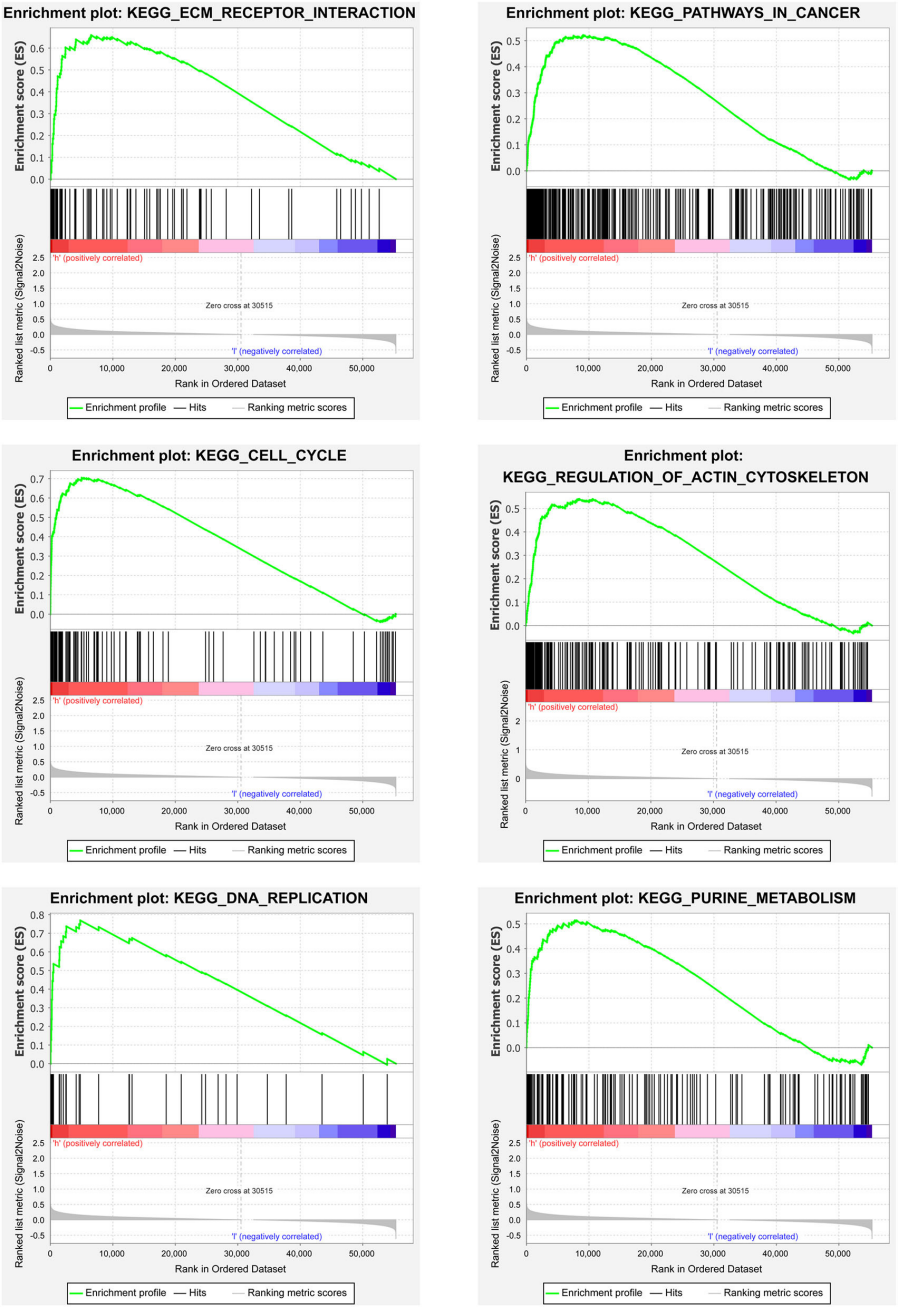
Gene set enrichment analyses (GSEA) analyses between the two risk pRCC groups divided by the lncRNAs related to ferroptosis in TCGA.

We then carried out the GSEA analysis. The results revealed that the majority of the prognostic signature based on the ferroptosis-related lncRNAs regulated tumor-related pathways, such as ECM receptor interaction, cell cycle, pathways in cancer, regulation of actin cytoskeleton, RNA replication, and purine metabolism (Figure 6).

### Immunity Analyses

**Figure 7:**
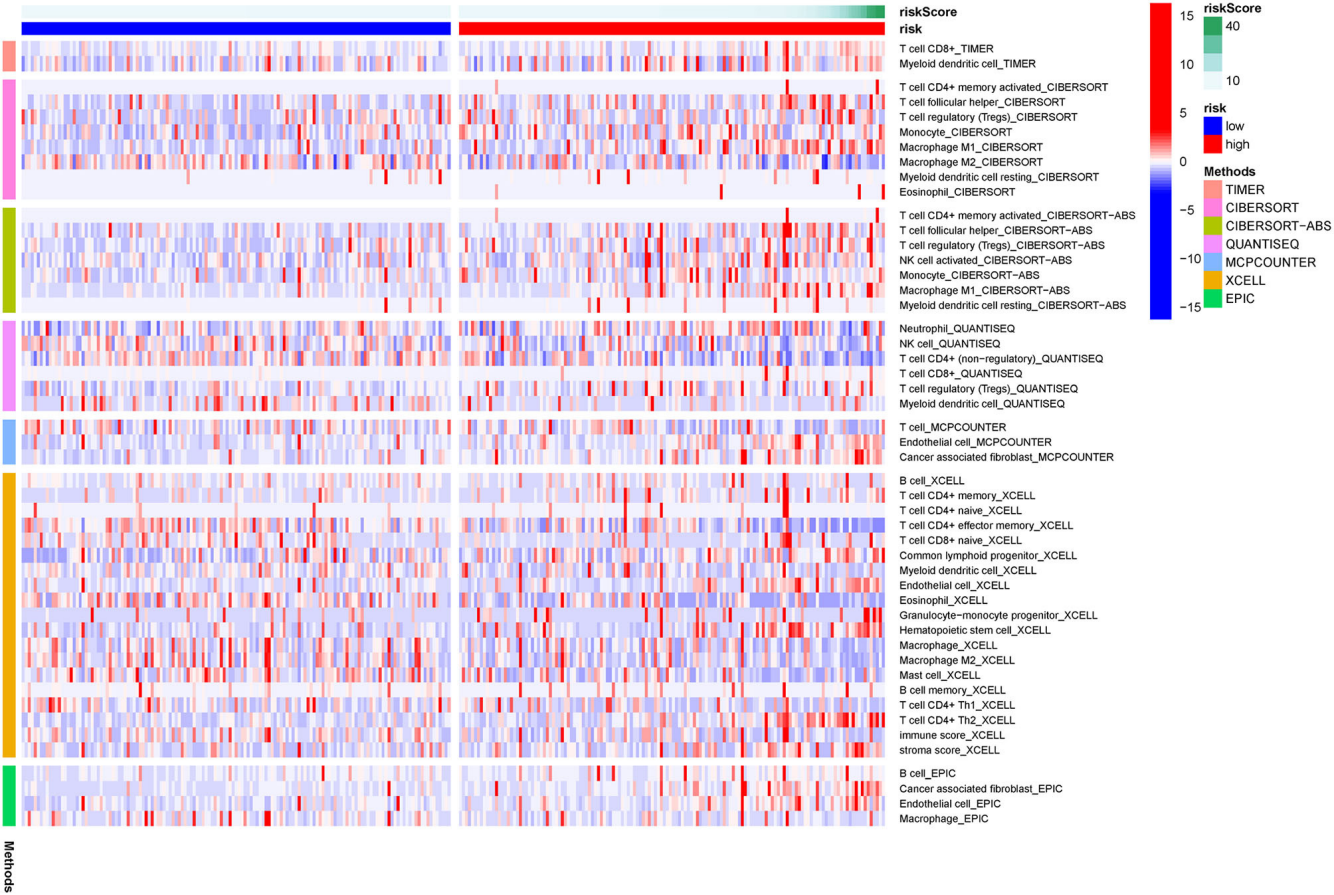
A heatmap of immune responses according to Tumor Immune Estimation Resource (TIMER) algorithms, CIBERSORT, QUANTISEQ, XCELL, MCP counter, and Estimating the Proportion of Immune and Cancer cells (EPIC) between the high- and low-risk pRCC groups.

**Figure 8:**
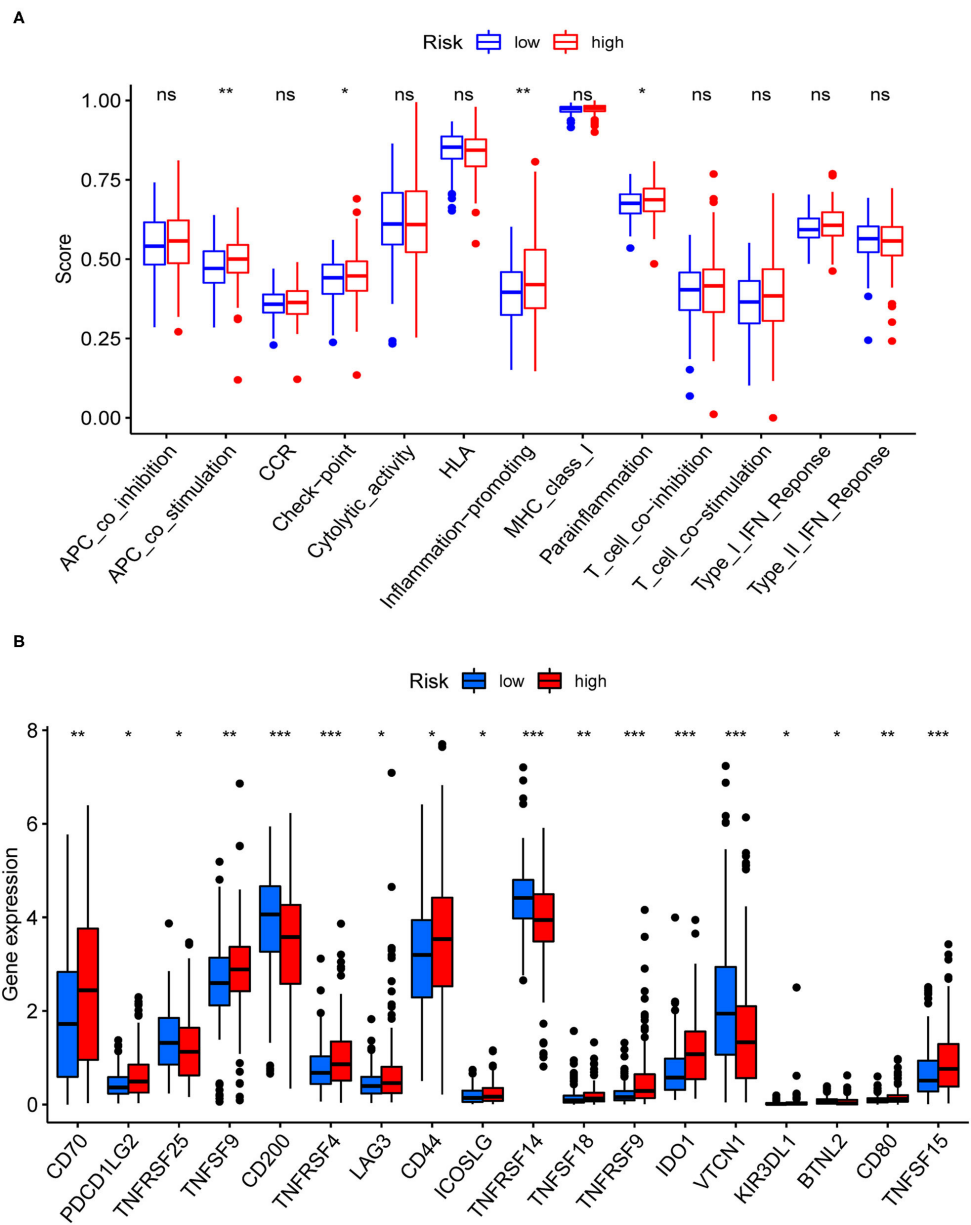
Immune-related analyses for the patients with pRCC from TCGA. **(A)** Correlation analysis for immune cell subpopulations between the two pRCC groups. **(B)** The expression levels of immune checkpoint genes between the two pRCC groups. *p < 0.05; **p < 0.01; ***p < 0.001; ns = non-significant.

Figure 7 showed the results of immune responses analyses, which were based on TIMER algorithms, CIBERSORT, QUANTISEQ, XCELL, MCP counter, and EPIC. The results of ssGSEA analyses of pRCC, which showed the correlation between immune cell subpopulations and related functions, revealed that APC co-stimulation, T cell functions such as checkpoint (inhibition), inflammation promoting, and para inflammation were significantly different between the two groups with different risks (Figure 8A). Since the checkpoint inhibitor-based immunotherapies are important in clinic, we further looked at the differences in immune checkpoint expression levels between the two risk groups. As shown in Figure 8B, it could be easily found that the expression levels of PDCD1LG2 (PD-L2), LAG3, IDO1 among others were substantially different between the two risk pRCC groups.

## Discussion

Ferroptosis may help to remove faulty cells while also overcoming cancer cell resistance to treatment (30). As a result, it has the potential to be a new tumor therapy strategy. Using the TCGA-pRCC data, we first discovered a new prognostic signature composed of identified ferroptosis-related lncRNAs. We investigated the functions of immune infiltrating cells and immune checkpoint inhibitors (ICIs) in the prognosis of pRCC since it is characterized by different immune microenvironments (6). In the ferroptosis signaling pathways, the findings of this research revealed a potential biomarker and therapeutic target.

Our analyses uncovered 56 ferroptosis-related DEG. KEGG results further revealed that the 56 genes mainly participated in cellular responses to chemical stress, response to oxidative stress, response to iron ion, response to oxygen levels, and so on. Ferroptosis is found mainly associated with oxidative stress, iron ion imbalance, and GSH homeostasis (31). Disturbed iron homeostasis was reported might lead to the iron-dependent form of cell death, ferroptosis (32). And, intracellular iron overload is the key to initiating ferroptosis (33). Depletion of cellular iron using iron chelators completely suppresses ferroptosis (34). P53 also sensitizes cells to ferroptosis by suppressing the transcription of SLC7A11 under the chemical stress, and thereby repressing the uptake of cystine in tumor cells (35). Activating the oxidative stress response gene Nrf2 could avoid ferroptosis (36), hence Nrf2 has been reported as a key gene of oxidative stress response which prevents ferroptosis. Iron chelators could prevent iron from transferring electrons to oxygen to inhibit ferroptosis so that reducing the ROS production (37). Overall, 5 lncRNAs related to ferroptosis were found could serve as independent prognosis factors for pRCC in this study. Recently, several studies also pointed out that the five identified lncRNAs were related to cancers. AC099850.3 was newly found to influence the acetylation level in non-small cell lung cancer (38). Also, it was closely associated with decreased survival of patients 0diagnosed with tongue cancer (39). LncTAM34A-mediated increase in the expression level of miR34a is sufficient to drive the appropriate cellular responses to stress stimuli (40). LncTAM34A was also reported to be a favorable prognostic factor of endometrial cancer (41), while it was also a negative factor for out risk signature. In addition, linc00462 promotes the invasiveness of pancreatic cancer cells *via* regulating the miR-665/TGFBR1-TGFBR2/SMAD2/3 pathway (42), hence it could promote cell progression (43). Moreover, cervical cancer patients with lower levels of FOXD2-AS1 had a higher OS rate than those with higher levels of FOXD2-AS1 (44). LINC02535 has been reported that could regulate DNA damage repair by combining with PCBP2 (45), and could promote the growth of cancer cells (46). So far, no researches have been done on the function of lncRNAs associated with ferroptosis in pRCC prognosis. Such discoveries in this study may offer crucial insight into cancer control in the future.

In this study, the differently expressed ferroptosis-associated lncRNAs were utilized to divide patients into two groups, in order to investigate their possible involvement in pRCC in this research. Ferroptosis coupled with ICIs was found could enhance anticancer efficacy synergistically, even in the tumors with resistance to ICIs (47). Only a few researches have looked at the connection between ICI and ferroptosis. MicroRNA (miRNA) and lncRNA are increasingly thought to have a role in ferroptosis control. MiRNA has been shown to disrupt ferroptosis by modulating the expression of Nrf2, whereas Nrf2 has been shown to limit iron absorption, decreasing the generation of ROS (48–50). Meanwhile, miRNA participates in the regulation of the transit, storage, use, and absorption process of iron. Several novel ferroptosis regulating factors, such as P53, SLC7A11, and the ACSL4 pathway, have been identified in recent years. Surprisingly, lncRNA is involved in the regulation of these factors' expression levels (51).

As we all know, ferroptosis is a novel type of cell death that has the potential to revolutionize tumor therapy. Many important questions, such as the relationship between ferroptosis and other cell deaths, as well as host immunogenicity, remain unanswered. As a result, this research looked at ferroptosis biomarkers that may be used to predict pRCC prognosis and, therefore, guide therapy options. Nonetheless, we need to test our signature characteristics with various cohorts. The trustworthiness of our findings cannot be completely assured since they were not verified using pRCC samples in clinic. Our results should also be used with care due to the limited clinical evidence. The prognostic prediction model established in this research, in general, requires further validation.

## Conclusion

Specific ferroptosis-related lncRNAs could be used to predict the prognosis of pRCC.

## Data Availability Statement

The datasets presented in this study can be found in online repositories. The names of the repository/repositories and accession number(s) can be found in the article/Supplementary Material.

## Author Contributions

Conceptualization and validation was done by YM and XT. Data curation was done by YC and FJ. Formal analysis was done by YC, FJ, and YX. Methodology was performed by YX and FJ. Supervision was analyzed by FJ and XW. Writing—original draft was done by YC and YX. Writing—review and editing was done by XW, YM, and XT. All authors contributed to the article and approved the submitted version.

## Conflict of Interest

The authors declare that the research was conducted in the absence of any commercial or financial relationships that could be construed as a potential conflict of interest. The reviewer JL declared a shared affiliation, though no other collaboration, with the authors to the handling Editor.

## Publisher's Note

All claims expressed in this article are solely those of the authors and do not necessarily represent those of their affiliated organizations, or those of the publisher, the editors and the reviewers. Any product that may be evaluated in this article, or claim that may be made by its manufacturer, is not guaranteed or endorsed by the publisher.

## Supplementary Material

Supplementary Figure 1 The risk scores of papillary renal cell carcinoma (pRCC) patients with different treatment outcome.

Supplementary Figure 2 The five ferroptosis-related expression levels of long non-coding RNAs (lncRNAs) in different treatment outcome in pRCC.

Supplementary Figure 3 Survival analysis of patients with pRCC. **(A)** Survival analysis of patients with pRCC based on the disease-specific survival (DSS) and the expression levels of five identified lncRNAs. **(B)** Survival analysis of patients with pRCC based on the overall survival (OS) and the expression levels of five identified lncRNAs.

Supplementary Table 1 The 382 ferroptosis-related genes downloaded from FerrDb.

Supplementary Table 2 Clinical information of papillary renal cell carcinoma (pRCC) patients from The Cancer Genome Atlas (TCGA) database.

Supplementary Table 3 The 56 differentially expressed gene (DEG) related to ferroptosis.

Supplementary Table 4 The 945 differentially expressed ferroptosis-related long non-coding RNAs (lncRNAs).

Supplementary Table 5 Results of univariate Cox analysis on the differentially expressed ferroptosis-related lncRNAs along with overall survival of pRCC samples.

## Abbreviations

RCC: renal cell carcinoma
pRCC: papillary RCC
ccRCC: clear cell RCC
chRCC: chromophobe renal cell carcinoma
TCGA: The Cancer Genome Atlas
OS: overall survival
ROS: reactive oxygen species
lncRNA: long non-coding RNA
GO: Gene Ontology
KEGG: Kyoto Encyclopedia of Genes and Genomes
GSEA: Gene set enrichment analyses
TIMER: Tumor Immune Estimation Resource
EPIC: Estimating the Proportion of Immune and Cancer cells
ssGSEA: single-sample GSEA
ROC: the receiver operating characteristic
DCA: the decision curve analysis
EC: endometrial cancer
ICI: immune checkpoint inhibitor.
