# Supplementary figures and images for: Identification of the Ferroptosis-Related Long Non-Coding RNAs Signature to Improve the Prognosis Prediction in Papillary Renal Cell Carcinoma
Source: Front Surg. 2022 Mar 4;9:741726. doi: 10.3389/fsurg.2022.741726 (PMC8930926; doi:10.3389/fsurg.2022.741726)

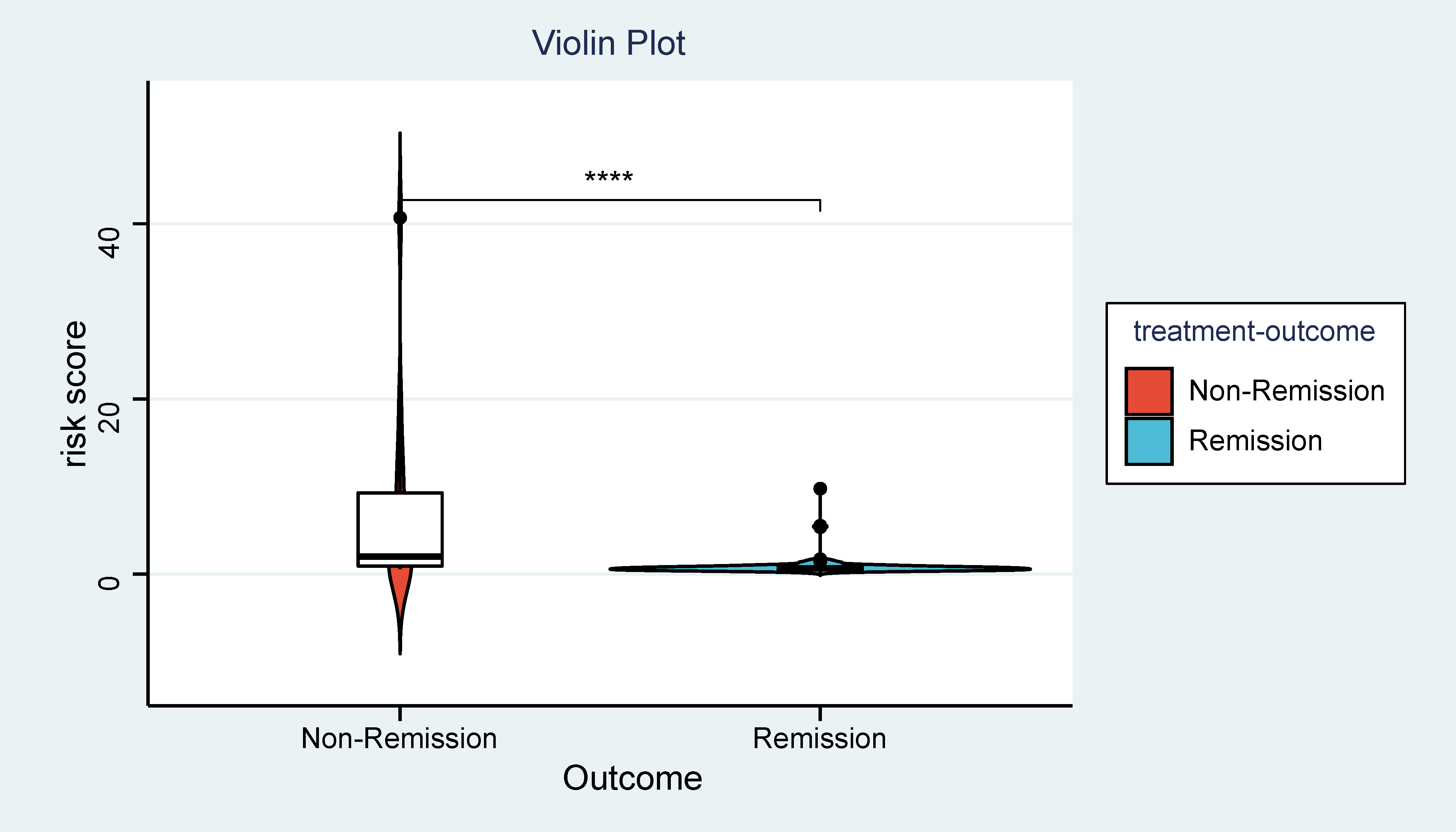

Supplement: Supplementary Figure 1 — The risk scores of papillary renal cell carcinoma (pRCC) patients with different treatment outcome. [file Image_1.TIF]

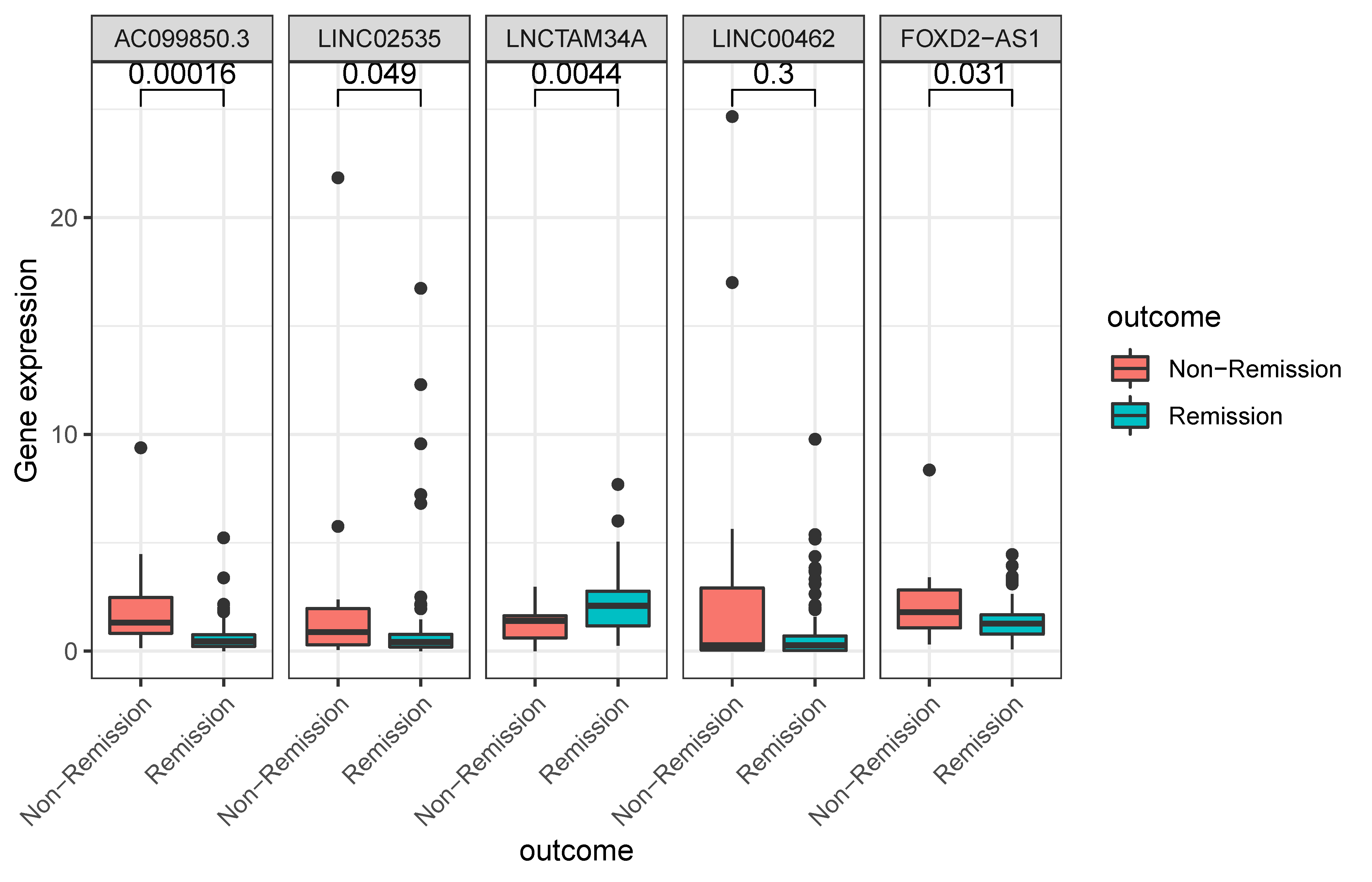

Supplement: Supplementary Figure 2 — The five ferroptosis-related expression levels of long non-coding RNAs (lncRNAs) in different treatment outcome in pRCC. [file Image_2.TIF]

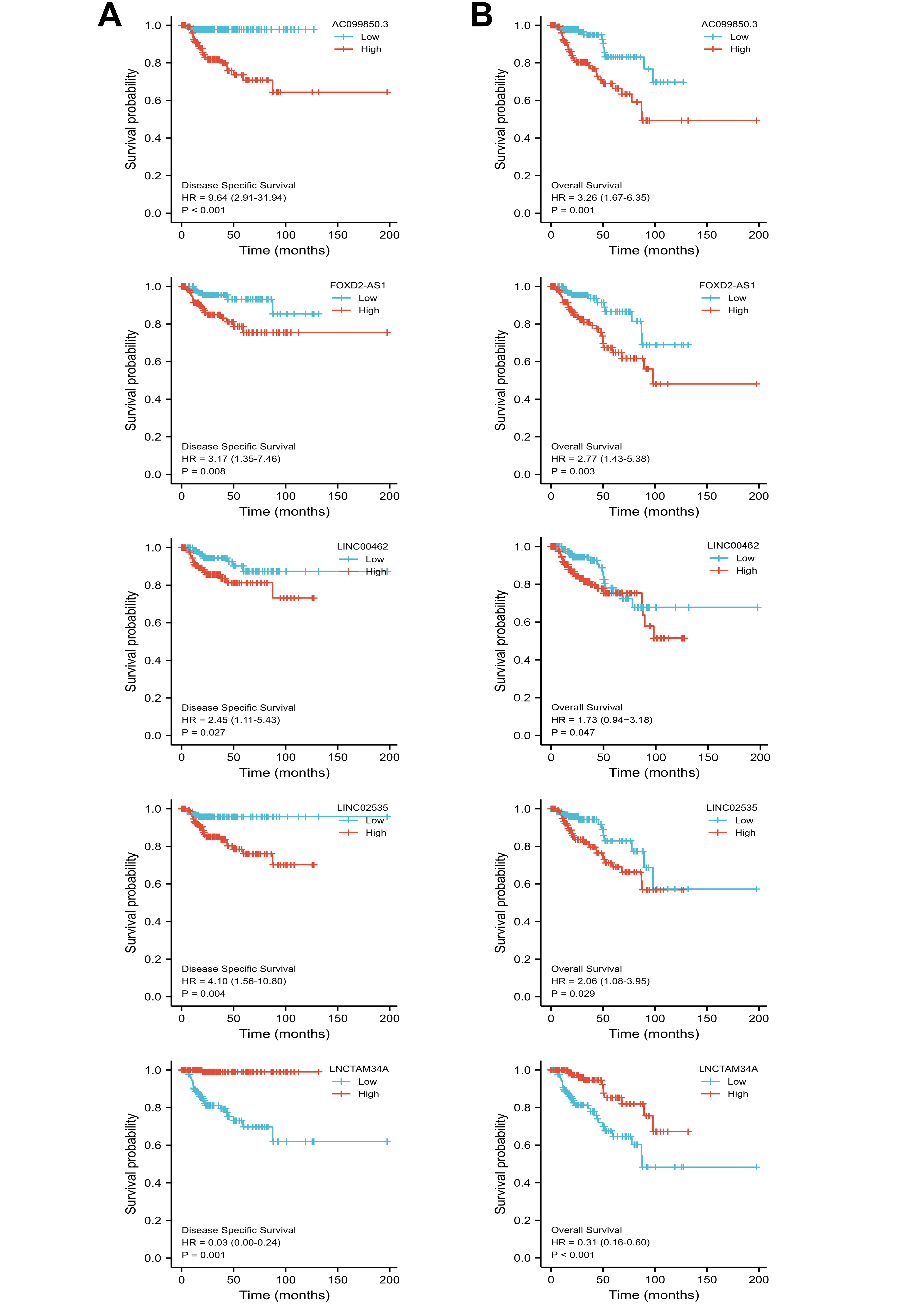

Supplement: Supplementary Figure 3 — Survival analysis of patients with pRCC. (A) Survival analysis of patients with pRCC based on the disease-specific survival (DSS) and the expression levels of five identified lncRNAs. (B) Survival analysis of patients with pRCC based on the overall survival (OS) and the expression levels of five identified lncRNAs. [file Image_3.TIF]
